# Evaluation of different methods of transoral minimally invasive surgery for supraglottic laryngeal carcinoma

**DOI:** 10.1002/cnr2.2077

**Published:** 2024-08-08

**Authors:** Lingwa Wang, Qi Zhong, Fan Yang, Lizhen Hou, Hongzhi Ma, Ling Feng, Shizhi He, Yifan Yang, Jugao Fang, Ru Wang

**Affiliations:** ^1^ Department of Otolaryngology–Head and Neck Surgery, Beijing Tongren Hospital Capital Medical University Beijing People's Republic of China; ^2^ Department of Otolaryngology–Head and Neck Surgery, Beijing Anzhen Hospital Capital Medical University Beijing People's Republic of China

**Keywords:** laryngeal function, prognosis, recurrence, supraglottic laryngeal carcinoma, transoral minimally invasive surgery, transoral robotic surgery

## Abstract

**Background and objectives:**

To analyze oncological and functional results of transoral minimally invasive surgery (TMIS) for supraglottic laryngeal carcinoma (SGLC), and investigate independent prognostic factors.

**Methods:**

Seventy SGLC patients treated with TMIS were included. The overall survival (OS), recurrence‐free survival (RFS), and postoperative functions were analyzed.

**Results:**

Sixty‐two patients were early‐stage (Tis, T1, and T2) and eight patients were T3. Eleven patients received preoperative induction chemotherapy (IC). Sixty patients received transoral laser microsurgery (TLM), and 10 patients received transoral robotic surgery (TORS). Fifty‐eight patients were scored Grade‐1 by water swallow test, and 49 patients were scored Grade 0 by grade, roughness, breathiness, asthenia, strain. The 1, 3, and 5 year OS of all were 95.450%, 84.877%, and 78.026%, and RFS were 89.167%, 78.052%, and 75.451% respectively. Kaplan–Meier survival analysis showed N stage and clinical stage were associated with OS, smoking, clinical stage, surgical margins, and Ki‐67 index were associated with RFS. There were no significant differences in preoperative IC or direct surgery, TLM, or TORS. Cox analyses showed smoking and surgical margins were independent prognosis factors for RFS.

**Conclusions:**

The positive margin, Ki‐67 index ≥40% and P53(+)&Ki‐67 index ≥40% are worse factors affecting recurrence for SGLC patients. Both smoking and surgical margins are independent prognostic factors affecting recurrence.

## 1 INTRODUCTION

Laryngeal cancer is the second most prevalent malignancy of the respiratory tract, and its incidence has been steadily increasing over the years. According to global statistics from 2020, there were approximately 180 000 new cases of laryngeal cancer reported, leading to approximately 99 000 deaths.^1^ Among the different types of laryngeal cancer, supraglottic laryngeal carcinoma (SGLC) accounts for around 35%–40% of cases.^2^ Despite having a lower incidence compared to other subtypes, SGLC often presents with insidious onset, atypical early symptoms, and an increased propensity for cervical lymph node metastasis.^2^, ^3^

The management of early‐stage SGLCs typically involves open supraglottic laryngectomy, transoral minimally invasive surgery (TMIS), and radiotherapy.^4^ In recent years, transoral approaches such as transoral laser microsurgery (TLM) and transoral robotic surgery (TORS) have gained increasing popularity due to their potential for good oncologic outcomes while reducing surgical complications and preserving functional integrity in early‐stage supraglottic carcinomas.^4^ These minimally invasive techniques offer advantages such as improved visualization, precise tumor resection, reduced morbidity, and enhanced postoperative functional outcomes.^5^, ^6^ However, despite growing evidence on the efficacy of TMIS, there is still a lack of well‐defined prognostic factors for patients undergoing these procedures.

Therefore, the aim of this study is to analyze both the oncological and functional outcomes of TMIS in the treatment of SGLC and identify potential independent prognostic factors. By thoroughly assessing the impact of TMIS on disease control and functional preservation, we aim to provide valuable insights into the effectiveness of these minimally invasive techniques in managing early‐stage SGLCs.

To achieve our objectives, a comprehensive analysis will be conducted using a combination of clinical data, follow‐up records, and patient‐reported outcomes. We will explore various factors such as tumor characteristics, patient demographics, treatment modalities, and postoperative complications to determine their influence on both oncological outcomes (e.g., disease recurrence, overall survival) and functional outcomes (e.g., voice quality, swallowing function) in SGLC patients undergoing TMIS.

The findings from this study have the potential to contribute significantly to the understanding and optimization of treatment strategies for early‐stage SGLC. By identifying independent prognostic factors associated with TMIS outcomes, we can enhance patient selection criteria, refine surgical techniques, and improve overall treatment outcomes. Additionally, this research may pave the way for personalized approaches in managing SGLC, leading to improved patient care and better long‐term survival rates.

In conclusion, this study aims to provide a comprehensive evaluation of the oncological and functional results of TMIS in the management of SGLC. By investigating potential prognostic factors, we strive to enhance our understanding of the disease and improve treatment outcomes for patients. The results of this study will contribute to the existing literature and guide future research efforts in optimizing the therapeutic approach for early‐stage SGLC.

## 2 MATERIALS AND METHODS

### 2.1 Study design and participants

This retrospective study included a total of 90 patients diagnosed with SGLC who underwent TMIS at Beijing Tongren Hospital and Beijing Anzhen Hospital, affiliated with Capital Medical University, between February 2008 and December 2021. The study was conducted in accordance with the principles outlined in the Declaration of Helsinki.

### 2.2 Inclusion and exclusion criteria

Detailed demographic, clinical, and pathological data were collected from a subset of 70 patients who met the following criteria: histologically confirmed diagnosis of SGLC, undergone TMIS as the primary treatment modality, complete clinical and follow‐up data available. All demographic, clinical, and pathological data were collected and recorded for analysis. This included information on patient characteristics (age, gender), clinical presentation, tumor characteristics, surgical procedure details, and postoperative outcomes. Twenty patients with incomplete clinical and follow‐up data were excluded from the analysis.

### 2.3 Evaluation of tumor characteristics

Prior to the surgical procedure, preoperative laryngoscopy was performed to evaluate the tumor characteristics including location, size, relationship with surrounding tissues, and suitability for TMIS. Under the guidance of a laryngoscope, the tumor was completely exposed to ensure visual field coverage of all tumor boundaries. A safe margin of at least 5 mm was meticulously preserved to secure the surgical margins and maintain a margin of safety during the procedure.

### 2.4 Pathological examination

Routine pathological examination of surgical margins was performed intraoperatively to confirm the safety of resection margins. Additionally, selective neck lymph node dissection was performed based on the extent of the lesion and neck examination, with the removal of the local lesion. Tumor staging was determined according to the 8th edition of the American Joint Committee on Cancer staging manual, taking into account variables such as tumor size, depth of invasion, and lymph node involvement.

### 2.5 Treatment regimen

TMIS served as the primary treatment modality for all included patients. TMIS, which encompasses TLM and TORS, has gained significant recognition for its ability to achieve favorable oncologic outcomes, minimize surgical complications, and preserve functional integrity in early‐stage supraglottic carcinomas. The specific techniques and procedures utilized during TMIS were carried out by experienced surgeons following established protocols and guidelines.

### 2.6 Functional and oncological evaluation

Functional outcomes, pathological results, and recurrences were carefully examined after the surgery. Swallowing function was evaluated on the basis of the water swallow test (WST), and phonation functions was evaluated by GRBAS scale (grade, roughness, breathiness, asthenia, strain).^7^ Immunohistochemical staining of Ki67 and p53 was performed on the postoperative pathological tissues. Positive Ki67 or p53 expression was determined when nuclei exhibited a brownish‐yellow color, with the Ki‐67 index calculated as the percentage of positive cells, and p53(+) defined as >10% positive cells.

### 2.7 Follow‐up

All patients included in the study were followed up through clinic visits or telephone contact until the data cutoff date (June 30, 2022) to assess their long‐term outcomes. The follow‐up period varied for each patient depending on their individual timeline from surgery. During the follow‐up period, the overall survival (OS) and recurrence‐free survival (RFS) of all patients were analyzed.

OS was defined as the time from the date of surgery to either the occurrence of death or the date of the last documented follow‐up. In other words, OS represents the length of time a patient survived from the initial surgery until any cause of death occurred or until the last follow‐up visit. RFS was computed from the date of surgery until the occurrence of disease recurrence, or the date of the last follow‐up contact. RFS measures the duration of time that a patient remained free from disease recurrence after the initial surgery.

### 2.8 Statistical analyses

The Kaplan–Meier (KM) method was used to draw survival curves, and the log‐rank test was used to compare the difference. Univariate and multivariate Cox regression analyses were performed to identify potential independent prognostic factors associated with OS and RFS. Variables significant on Cox univariate analysis (*p* < 0.05) were included in the multivariate analysis. All statistical analyses were carried out using SPSS (version 26). *p* < 0.05 were considered statistically significant.

## 3 RESULTS

### 3.1 Patient characteristics

Detailed clinical and pathological information of all 70 patients were presented in Table 1. There were 58 males and 12 females, with a median age of 64 (ranged from 38 to 81 years old). Of the 70 cases, 47 patients (67.1%) had a smoking history, and 38 patients (54.3%) had a drinking history. The T stage was early‐stage (Tis, T1, and T2) for 62 patients (88.6%), and T3 for eight patients (11.4%) (six patients received preoperative induction chemotherapy (IC), and two patients declined open surgery because of general poor tolerance). Surgical margins were positive in six patients (8.6%). Patients with positive surgical margins are typically recommended to undergo comprehensive treatment, including postoperative radiotherapy and/or combined chemotherapy. Close follow‐up of the patients is also conducted to monitor their condition and response to treatment. A total of 11 patients (15.7%) (6 patients were at T3 stage, 3 patients were at T2 stage, and 2 patients were at T1 stage) received preoperative IC, and primary tumor volume was significantly reduced (≥30%). So these patients underwent TMIS. Sixty patients (85.7%) were treated with TLM, and 10 patients (14.3%) were treated with TORS. Four patients (5.7%) underwent temporary tracheostomy, and they were extubated successfully 1–3 months after surgery. Forty‐four patients (62.9%) had a nasogastric feeding tube postoperatively, and it was removed 5–30 days postoperatively.

**TABLE 1:** Clinical and pathologic information on the patients.

Characteristics	No. of patients (%)
Sex	
Male	58 (82.9)
Female	12 (17.1)
Age	
≥60	55 (78.6)
<60	15 (21.4)
Smoking	
Yes	47 (67.1)
No	23 (32.9)
Drinking	
Yes	38 (54.3)
No	32 (45.7)
T stage	
Tis	4 (5.7)
T1	30 (42.9)
T2	28 (40.0)
T3	8 (11.4)
N stage	
N0	60 (85.7)
N1	6 (8.6)
N2	4 (5.7)
M stage	
M_0_	100 (100)
Clinical stage	
Stage 0	4 (5.7)
Stage 1	28 (40.0)
Stage 2	22 (31.4)
Stage 3	12 (17.2)
Stage 4	4 (5.7)
Surgical margins	
Negative	64 (91.4)
Positive	6 (8.6)
P53	
Negative	41 (58.6)
Positive	29 (41.4)
Ki‐67 index (%)	
<40	31 (44.3)
≥40	39 (55.7)
Induction chemotherapy	
Yes	11 (15.7)
No	59 (84.3)
Surgical approaches	
TLM	60 (85.7)
TORS	10 (14.3)

Abbreviations: TLM, transoral laser microsurgery; TORS, transoral robotic surgery.

### 3.2 Functional outcomes

The swallow and pronunciation functions of all patients were evaluated by WST and GRBAS scale 6 months after surgery. According to WST, 58 patients (82.9%) were scored as Grade 1 (excellent) who can successfully swallow water once, 10 patients (14.3%) were scored as Grade 2 (good) who can swallow water twice or more without cough, and two patients (2.9%) were scored as Grade 3 (mild) who can swallow water once but with cough. According to GRBAS scale, 49 patients (70.0%) were scored as Grade 0 (normal) who had normal speech function, 19 patients (27.1%) were scored as Grade 1 (mild) who had minor hoarseness, one patient (1.4%) were scored as Grade 2 (moderate) who had hoarseness. Only one patient (1.4%) underwent total laryngectomy upon local tumor recurrence. The larynx preservation rate of the 70 SGLC patients was 98.6%.

### 3.3 Survival analysis

**FIGURE 1:**
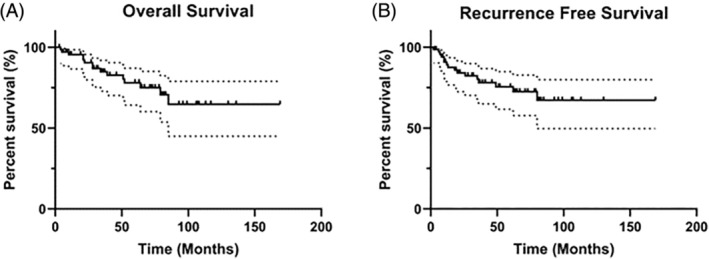
Survival analysis of all included supraglottic laryngeal carcinoma patients. (A) Analysis of the OS time. (B) Analysis of the RFS time. OS, overall survival; RFS, recurrence‐free survival.

The median follow‐up time was 41 months (ranged from 2 to 169 months.). Twenty‐five patients (35.7%) experienced recurrence or death during the follow‐up period, of which 16 patients (22.9%) recurred at 2–80 months after diagnosis (median, 18 months) and 15 patients (21.4%) died at 4–85 months (median, 28 months). The 1‐, 3‐, and 5‐year OS of all patients were 95.450%, 84.877%, and 78.026%, respectively, and the median OS time was 48.5 months (Figure 1A). The 1‐, 3‐, and 5‐year RFS were 89.167%, 78.052%, and 75.451%, respectively, and the median RFS time was 36.5 months (Figure 1B).

**FIGURE 2:**
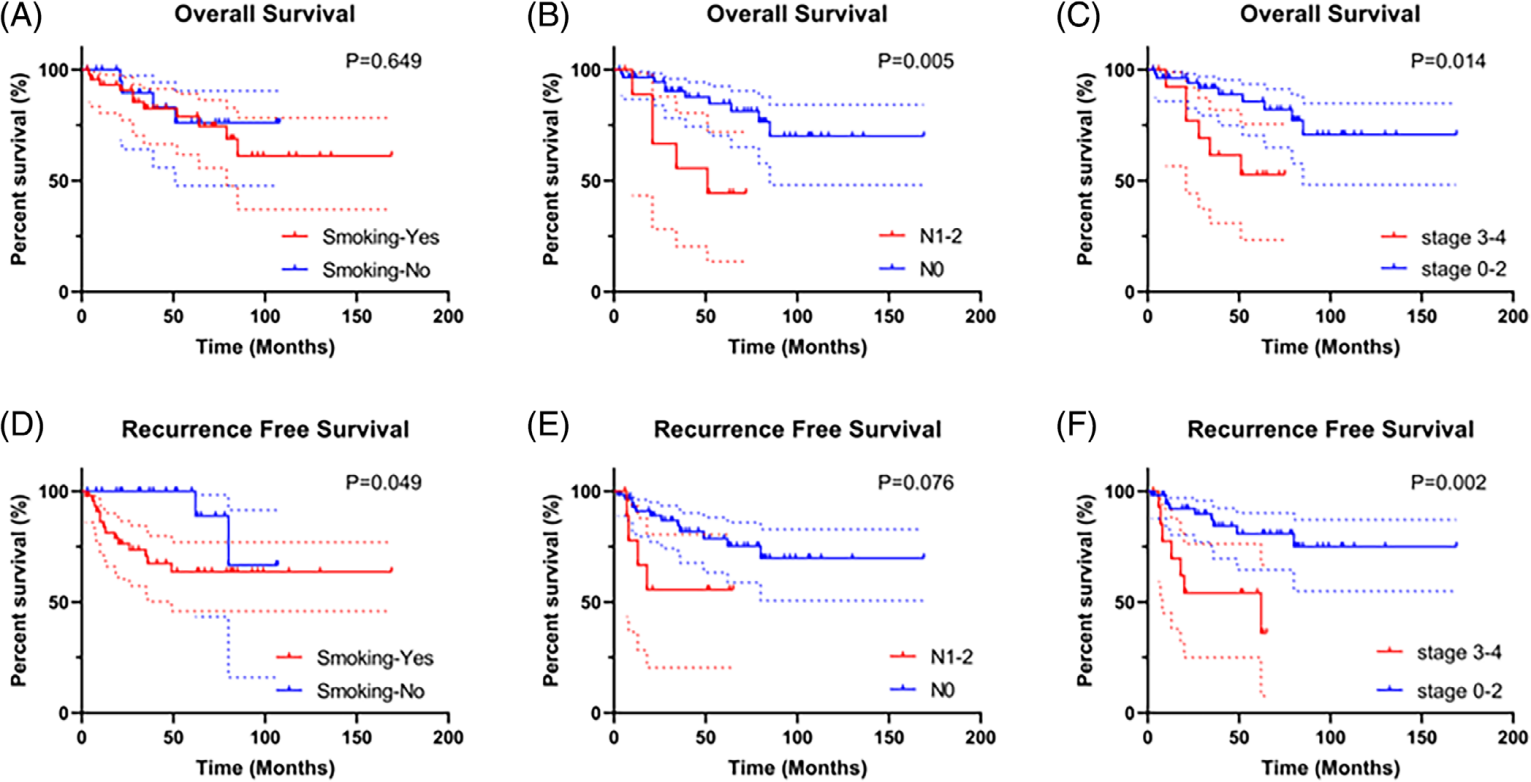
KM survival analysis of patients with different clinical subgroups. (A–C) OS times were compared between different smoking status, N stages and clinical stages. (D–F) RFS times were compared between different smoking status, N stages and clinical stages. KM, Kaplan–Meier; OS, overall survival; RFS, recurrence‐free survival.

**FIGURE 3:**
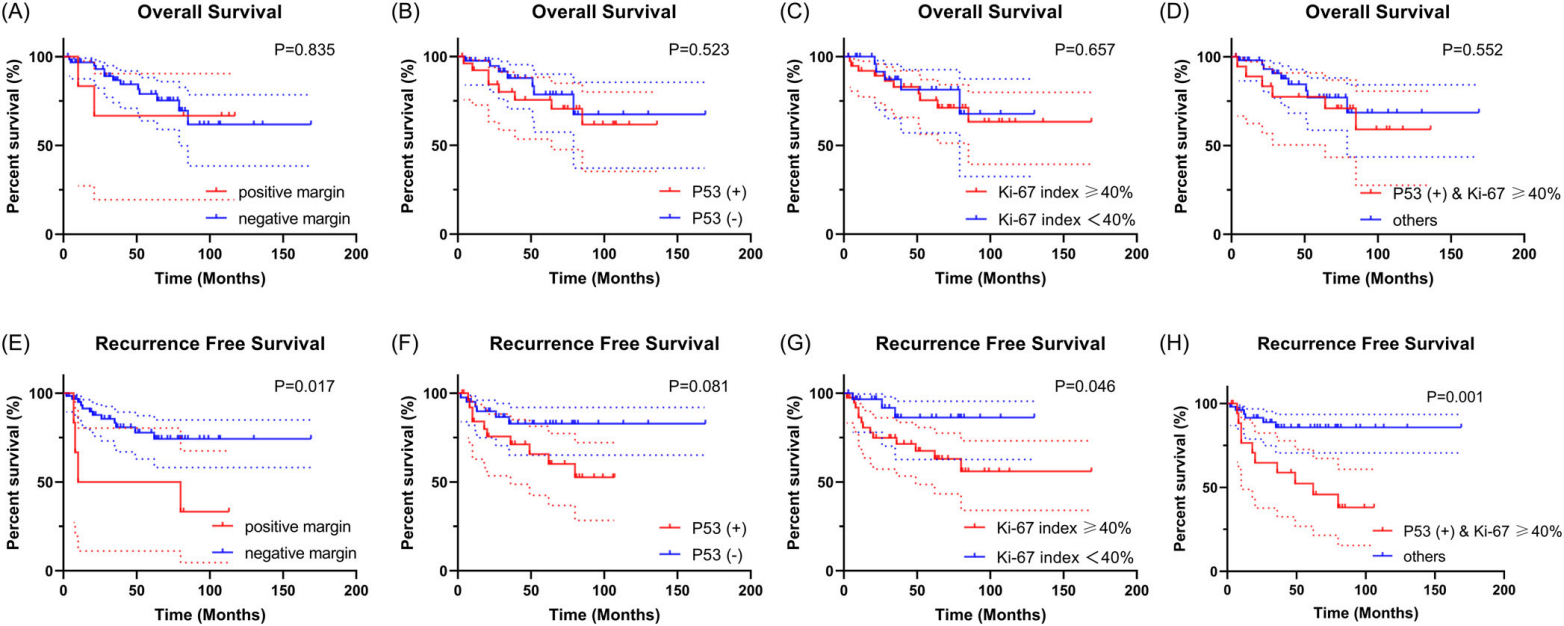
KM survival analysis of patients with different pathologic features. (A–D) OS times were compared between different surgical margins, P53 mutant and Ki‐67 index. (E–H) RFS times were compared between different surgical margins, P53 mutant and Ki‐67 index. KM, Kaplan–Meier; OS, overall survival; RFS, recurrence‐free survival.

**FIGURE 4:**
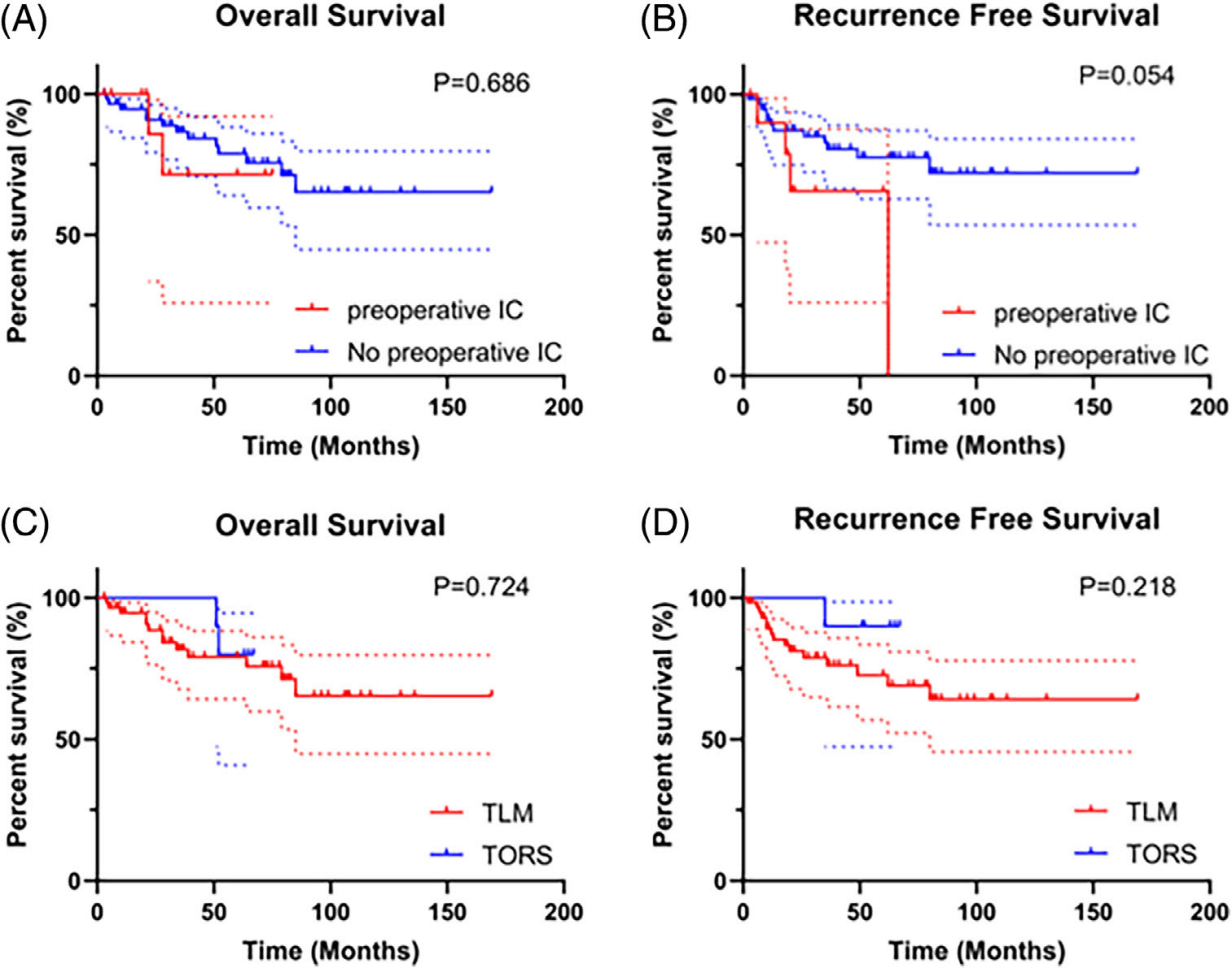
KM survival analysis of patients with different treatment approaches. (A) OS times were compared between preoperative IC subgroup and no preoperative IC subgroup. (B) RFS times were compared between preoperative IC subgroup and no preoperative IC subgroup. (C) OS times were compared between TLM subgroup and TORS subgroup. (D) RFS times were compared between TLM subgroup and TORS subgroup. IC, induction chemotherapy; KM, Kaplan–Meier; OS, overall survival; RFS, recurrence‐free survival; TLM, transoral laser microsurgery; TORS, transoral robotic surgery.

**FIGURE 5:**
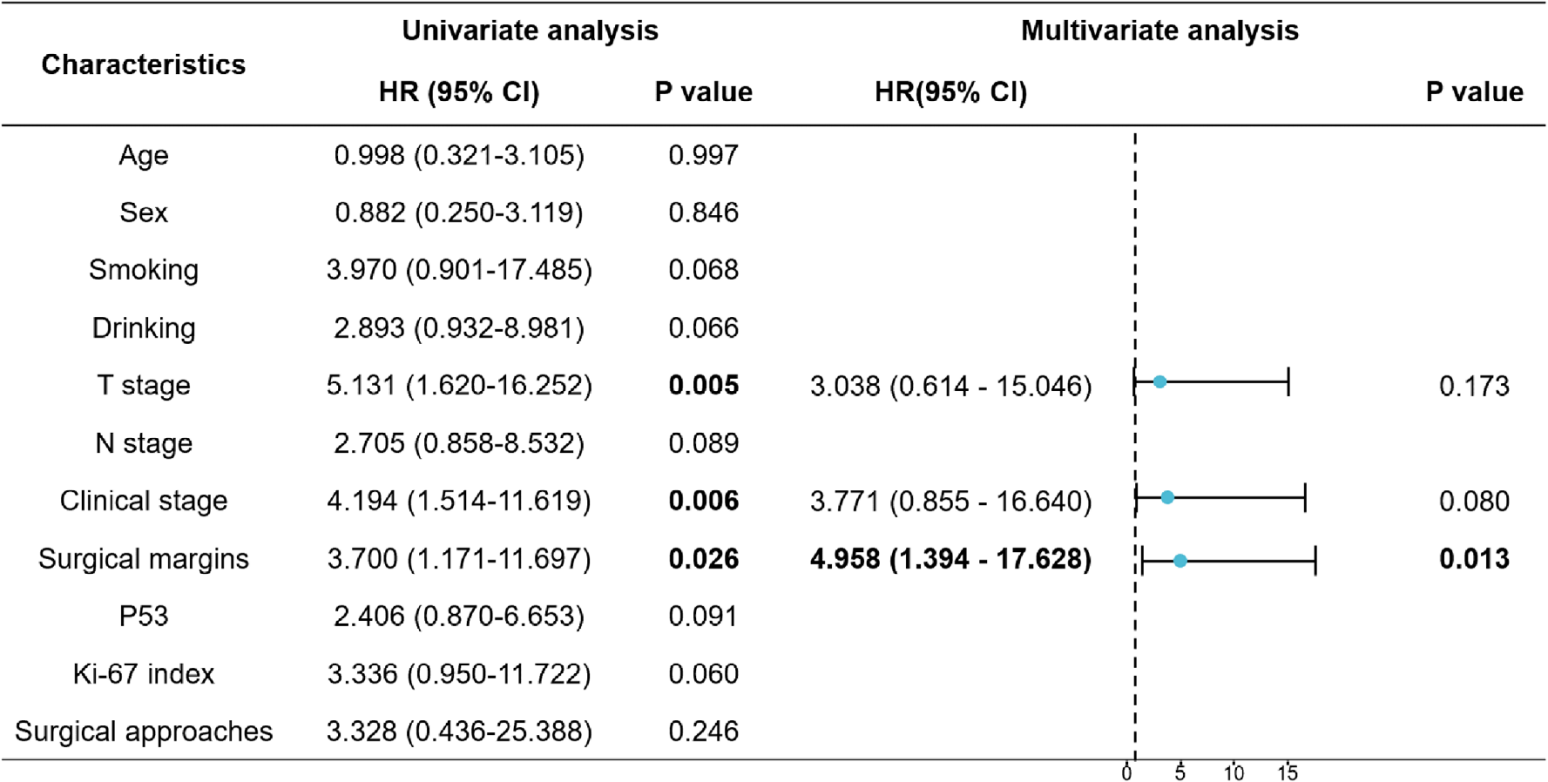
Forest plot for Cox regression analyses in RFS. RFS, recurrence‐free survival.

Univariate KM survival analysis showed that the OS of patients with lymph node metastasis were worse than patients without lymph node metastasis, the RFS of patients with smoking history were worse than patients without smoking history, and the OS and RFS of late‐stage patients were worse than early‐stage patients (*p* < 0.05) (Figure 2). Although there were no significant differences in surgical margins, P53 mutant, and Ki‐67 index for OS (*p* > 0.05), the RFS of patients with positive margin, Ki‐67 index ≥40% and P53(+)&Ki‐67 index ≥40% were worse (*p* < 0.05) (Figure 3). There were no significant differences between preoperative IC group and direct surgery group based on OS and RFS (*p* > 0.05) (Figure 4A,B). Similarly, the KM analysis showed no significant differences between TLM and TORS based on OS and RFS (*p* > 0.05) (Figure 4C,D). Additionally, Cox regression analyses were performed both for OS and RFS. Univariate and multivariate analyses identified surgical margins were independent prognosis factors for RFS in SGLC patients (*p* < 0.05) (Figure 5).

## 4 DISCUSSION

The mainstay of treatment options for early‐stage laryngeal cancer included open partial laryngectomy, TLM, TORS, or radiation therapy.^4^ Open partial laryngectomy has gradually decreased in popularity due to its impact on partial laryngeal function. However, TLM, TORS, and radiation therapy have shown superior functional outcomes in terms of voice quality and swallowing.^8^, ^9^ Our study findings are consistent with previous research that highlights the advantages of TLM and TORS over open laryngectomy. Karatzanis et al conducted a retrospective analysis comparing TLM and open laryngectomy, finding no significant differences in disease‐specific survival and local control between the two approaches. However, TLM had a lower incidence of complications and better functional results.^10^ Gökmen et al yielded similar results, supraglottic cancer patients treated with TLM had shorter hospitalization times, earlier transition to oral intake, less need for tracheotomy and better voice quality outcomes compared to the patients who underwent open laryngectomy.^8^ In accordance with these researches, 58 patients (82.9%) can successfully swallow without cough, and 49 patients (70.0%) had normal speech function after TMIS in our study. Temporary tracheostomy was performed in four patients (5.7%) and all were extubated as planned after surgery. The larynx preservation rate of the 70 SGLC patients was 98.6% except one patient underwent total laryngectomy upon tumor recurrence.

Transoral microelectrodes surgery (TOMES) is an emerging technique for supraglottic surgery, offering valuable insights into alternative approaches for managing supraglottic carcinoma. In a study conducted by Jorge Basterra et al, they assessed the postoperative functional and oncological outcomes of 83 patients with T1 to T3 laryngeal carcinomas who underwent TOMES. Their findings demonstrated several advantages of TOMES over TLM, including improved hemostasis, simpler handling, the ability to perform angled cuts, and provide a sense of touch, shorter operation time, and reduced equipment costs.^11^ Subsequently, Basterra extended the scope of their research and then consistently concluded that all tumors resectable with CO_2_ laser could be effectively resected with TOMES.^12^, ^13^ However, due to the limited number of patients treated with TOMES in our own study, we were unable to conduct meaningful statistical analyses or draw significant conclusions regarding its efficacy and outcomes when compared to other techniques. In future research endeavors, we aim to address these limitations by incorporating larger sample sizes or engaging in multicenter collaborations. By doing so, we will strive to include TOMES in our investigations and explore its comparative effectiveness and outcomes in managing supraglottic carcinoma.

The 5‐year OS of early stage supraglottic cancer patients after TMIS in different studies ranges from 62% to 84.2%, and the 5‐year RFS ranges from 72% to 90.2%.^5^, ^8^, ^14^, ^15^ Prognostic factors for OS and tumor recurrence in previous studies are not fully characterized. Doazan et al reported a multicenter observational study of 122 patients who underwent TORS, 28 patients died, and 14 patients recurred. The N classification was risk factor affecting local control and disease‐free survival by univariate analyses.^15^ Three patients died and five patients recurred of 19 supraglottic cancer patients treated with TLM in Gökmen ‘s study. And their results showed positive cervical lymph node was the most important survival factor.^8^ Dyckhoff et al's multivariable Cox regression model revealed no significant survival difference between patients with different margins and adjuvant treatment,^5^ Hans et al also pointed none of the histopathological index (T stage, N stage, tumor location, extracapsular spread, perineural, or lymphovascular invasion) proved to be independent prognostic factor.^16^ In our study, we aimed to include more specific pathological indicators. KM survival analysis revealed associations between OS and N stage and clinical stage, and between RFS and smoking history, clinical stage, surgical margins, P53 mutation, and Ki‐67 index. Additionally, Cox regression analyses identified surgical margins were independent prognosis factors for RFS in supraglottic cancer patients. Ki‐67 is a proliferation marker used to assess tumor cell activity and proliferative capacity. P53 is a tumor suppressor gene that plays a crucial role in regulating cell proliferation, apoptosis, and DNA repair processes. In our study, we observed a significant association between high Ki‐67 index, P53 mutant, and poor prognosis in SGLC patients. A high Ki‐67 index indicates active tumor cell proliferation, which may lead to faster tumor growth and metastasis. The functional loss of P53 may contribute to increased invasiveness. Therefore, Ki‐67 index and P53 mutant can be considered as important prognostic indicators to evaluate the risk of prognosis in patients and guide treatment decisions.

Regarding IC, it has been shown to be a beneficial treatment option for T3 or T4 laryngeal cancer patients seeking larynx preservation. IC can effectively reduce tumor volume without compromising survival, leading to improved rates of laryngeal function preservation and quality of life.^17^, ^18^ However, there is little report about application of IC in late‐stage laryngeal cancer, particularly supraglottic cancer. Only in Carta et al's series, platinum‐based IC was performed in two T2 supraglottic cancer patients, but volume of tumor did not change after IC.^19^ Our study included 11 patients who received IC, resulting in a reduction in tumor volume. Subsequently, these patients underwent TLM immediately after IC. Postoperative pathology showed no evidence of carcinoma in five cases, carcinoma in situ in four cases and moderately differentiated squamous cell carcinoma in two patients. Preoperative IC may be a good choice to degrade the tumor and preserve organ function.

For early supraglottic cancer, both TLM and TORS have been increasingly utilized in clinical. Multicenter studies have shown the use of TLM and TORS had advantages of less invasive, more safety and equivalent surgical margins compared to open surgery for selected supraglottic cancer,^5^, ^15^, ^20^ while there is a lack of research comparing TORS with TLM.^8^, ^20^ Weinstein et al pointed TORS had advantages of three‐dimensional visualization, tremor filtration, and excellent hemostasis.^20^ Hussain et al retrospectively assessed the oncologic outcomes of 84 supraglottic cancer patients (19 patients treated with TORS and 65 patients treated with TLM), this analysis showed TORS group had excellent local tumor control but no significant difference in disease‐free survival compared to TLM.^21^ In our research, we included 10 patients treated with TORS and 60 patients treated with TLM, the results showed there were no significant differences between TLM and TORS based on OS and RFS. Unfortunately, the number of patients included was limited, and more patients are needed to gain more insights in the future.

This study has several strengths. Firstly, it includes a relatively large number of patients who underwent TMIS for SGLC, allowing for meaningful analyses and generalizability of the findings within this patient population. Additionally, the inclusion of specific pathological indicators such as Ki‐67 index and P53 mutation provides valuable prognostic information. However, there are also certain limitations to consider in this study. Firstly, the retrospective nature of the study introduces potential selection biases. The data collected may be influenced by factors such as incomplete medical records or missing data, which could affect the validity and reliability of the results. Additionally, as with any single‐center study, there may be limitations inherent to this study design. The findings and conclusions drawn from a single institution may not necessarily be representative of other institutions or diverse populations. Variations in patient demographics, healthcare practices, and treatment protocols across different centers may impact the generalizability of our results. Therefore, caution should be exercised when extrapolating the findings to broader populations. Furthermore, the sample size of the study might be relatively small. Incorporating multicenter collaborations in future research endeavors would enhance the validity, applicability, and clinical relevance of the findings.

## 5 CONCLUSIONS

In conclusion, our study underscores the favorable functional outcomes and oncologic efficacy of TMIS (TLM and TORS) compared to open surgery for early‐stage SGLC. The inclusion of specific pathological indicators expands our understanding of prognostic factors in this patient population. The positive margin, Ki‐67 index ≥40%, and P53(+)&Ki‐67 index ≥40% are worse factors affecting recurrence for SGLC patients treated with TMIS by KM survival analysis. In addition, surgical margins are independent poor prognostic factors affecting recurrence by Cox analysis. Additionally, our findings support the potential benefits of IC in reducing tumor volume and preserving organ function. Besides, for late‐stage lesions, IC may be a good choice. While both TLM and TORS have demonstrated their advantages, further research with larger cohorts is necessary to directly compare the two approaches. The study has limitations due to potential selection biases, a single‐center design, and a small sample size. Future research should address these limitations by involving multiple centers and larger sample sizes for more reliable results. Overall, this study contributes to the existing knowledge base and may guide treatment decisions for SGLC patients undergoing TMIS.

## AUTHOR CONTRIBUTIONS

All authors contributed to the study conception and design. Material preparation and data collection were performed by Lingwa Wang, Qi Zhong, Fan Yang, Lizhen Hou, Hongzhi Ma, data analysis were performed by Lingwa Wang, Ru Wang, Ling Feng, Shizhi He, Qian Shi and Yifan Yang. The first draft of the manuscript was written by Lingwa Wang, Ru Wang and Jugao Fang, and all authors commented on previous versions of the manuscript. All authors read and approved the final manuscript.

## FUNDING INFORMATION

The Capital Health Research and Development of Special (No. 2022‐1‐2051); National Natural Science Foundation of China (No. 82002880).

## CONFLICT OF INTEREST STATEMENT

The authors have stated explicitly that there are no conflicts of interest in connection with this article.

## ETHICS STATEMENT

Approval of the research protocol by an Institutional Reviewer Board: The study was conducted according to the guidelines of the Declaration of Helsinki, and approved by the Institutional Review Board of the Beijing Tongren Hospital, Capital Medical University. All informed consent was obtained from the subjects or guardians. Registry and the registration No. of the study/trial: No.TREC2022‐KY018.R1.

## Data Availability

The data underlying this article are available in the airtlcle.

## References

[1] Sung H , Ferlay J , Siegel RL , et al. Global cancer statistics 2020: GLOBOCAN estimates of incidence and mortality worldwide for 36 cancers in 185 countries. CA Cancer J Clin. 2021;71(3):209‐249. 33538338 10.3322/caac.21660

[2] Tamaki A , Miles BA , Lango M , Kowalski L , Zender CA . AHNS series: do you know your guidelines? Review of current knowledge on laryngeal cancer. Head Neck. 2018;40(1):170‐181. 29076227 10.1002/hed.24862

[3] Sanabria A , Shah JP , Medina JE , et al. Incidence of occult lymph node metastasis in primary larynx squamous cell carcinoma, by subsite, T classification and neck level: a systematic review. Cancers. 2020;12(4):1059. 32344717 10.3390/cancers12041059 PMC7225965

[4] Patel TD , Echanique KA , Yip C , et al. Supraglottic squamous cell carcinoma: a population‐based study of 22,675 cases. Laryngoscope. 2019;129(8):1822‐1827. 30536822 10.1002/lary.27592

[5] Dyckhoff G , Warta R , Herold‐Mende C , Rudolph E , Plinkert PK , Ramroth H . An observational cohort study on 194 supraglottic cancer patients: implications for laser surgery and adjuvant treatment. Cancers. 2021;13(3):568. 33540592 10.3390/cancers13030568 PMC7867201

[6] Gorphe P . A contemporary review of evidence for transoral robotic surgery in laryngeal cancer. Front Oncol. 2018;8:121. 29721446 10.3389/fonc.2018.00121 PMC5915483

[7] Saraniti C , Ciodaro F , Galletti C , Gallina S , Verro B . Swallowing outcomes in open partial horizontal laryngectomy type I and endoscopic supraglottic laryngectomy: a comparative study. Int J Environ Res Public Health. 2022;19(13):8050. 35805718 10.3390/ijerph19138050 PMC9265323

[8] Gökmen MF , Büyükatalay ZÇ , Beton S , et al. Functional and oncological outcomes of open partial laryngectomy vs. transoral laser surgery in supraglottic larynx cancer. Turk Arch Otorhinolaryngol. 2020;58(4):227‐233. 33554197 10.5152/tao.2020.5573 PMC7846301

[9] Hrelec C . Management of Laryngeal Dysplasia and Early Invasive Cancer. Curr Treat Options Oncol. 2021;22(10):90. 34424405 10.1007/s11864-021-00881-w

[10] Karatzanis AD , Psychogios G , Zenk J , et al. Evaluation of available surgical management options for early supraglottic cancer. Head Neck. 2010;32(8):1048‐1055. 19953613 10.1002/hed.21289

[11] Basterra J , Reboll R , Zapater E . Eighty‐three cases of glottic and supraglottic carcinomas (stage T1‐T2‐T3) treated with transoral microelectrode surgery: how we do it. Clin Otolaryngol. 2011;36(5):500‐504. 22032451 10.1111/j.1749-4486.2011.02336.x

[12] Basterra J , Esteban F , Reboll R , Menoyo A , Zapater E . Transoral resection of supraglottic tumours using microelectrodes (54 cases). Eur Arch Otorhinolaryngol. 2014;271(9):2497‐2502. 24695940 10.1007/s00405-014-3002-x

[13] Basterra J , Oishi N , Alba JR , Zapater E . Microelectrodes and radiofrequency for transoral horizontal supraglottic laryngectomy in T3 tumors. Head Neck. 2023;45(1):283‐287. 36245292 10.1002/hed.27221

[14] Ambrosch P , Gonzalez‐Donate M , Fazel A , Schmalz C , Hedderich J . Transoral laser microsurgery for supraglottic cancer. Front Oncol. 2018;8:158. 29868479 10.3389/fonc.2018.00158 PMC5954241

[15] Doazan M , Hans S , Morinière S , et al. Oncologic outcomes with transoral robotic surgery for supraglottic squamous cell carcinoma: results of the French robotic surgery group of GETTEC. Head Neck. 2018;40(9):2050‐2059. 30051531 10.1002/hed.25199

[16] Hans S , Chekkoury‐Idrissi Y , Circiu MP , Distinguin L , Crevier‐Buchman L , Lechien JR . Surgical, oncological, and functional outcomes of transoral robotic supraglottic laryngectomy. Laryngoscope. 2021;131(5):1060‐1065. 32812245 10.1002/lary.28926

[17] Steuer CE , El‐Deiry M , Parks JR , et al. An update on larynx cancer. CA Cancer J Clin. 2017;67(1):31‐50. 27898173 10.3322/caac.21386

[18] Spector ME , Rosko AJ , Swiecicki PL , Chad Brenner J , Birkeland AC . From VA larynx to the future of chemoselection: defining the role of induction chemotherapy in larynx cancer. Oral Oncol. 2018;86:200‐205. 30409302 10.1016/j.oraloncology.2018.09.026

[19] Carta F , Mariani C , Sambiagio GB , et al. CO2 transoral microsurgery for supraglottic squamous cell carcinoma. Front Oncol. 2018;8:321. 30234007 10.3389/fonc.2018.00321 PMC6131582

[20] Weinstein GS , O'Malley BW Jr , Magnuson JS , et al. Transoral robotic surgery: a multicenter study to assess feasibility, safety, and surgical margins. Laryngoscope. 2012;122(8):1701‐1707. 22752997 10.1002/lary.23294

[21] Lechien JR , Fakhry N , Saussez S , et al. Surgical, clinical and functional outcomes of transoral robotic surgery for supraglottic laryngeal cancers: a systematic review. Oral Oncol. 2020;109:104848. 32534362 10.1016/j.oraloncology.2020.104848

